# Causal illusion in the core of pseudoscientific beliefs: The role of information interpretation and search strategies

**DOI:** 10.1371/journal.pone.0272201

**Published:** 2022-09-09

**Authors:** Marta N. Torres, Itxaso Barberia, Javier Rodríguez-Ferreiro

**Affiliations:** Grup de Recerca en Cognició i Llenguatge (GRECIL), Departament de Cognició, Desenvolupament i Psicologia de l’Educació, Secció de Processos Cognitius, Institut de Neurociències (INUB), Universitat de Barcelona (UB), Barcelona, Spain; University of Sydney, AUSTRALIA

## Abstract

The prevalence of pseudoscientific beliefs in our societies negatively influences relevant areas such as health or education. Causal illusions have been proposed as a possible cognitive basis for the development of such beliefs. The aim of our study was to further investigate the specific nature of the association between causal illusion and endorsement of pseudoscientific beliefs through an active contingency detection task. In this task, volunteers are given the opportunity to manipulate the presence or absence of a potential cause in order to explore its possible influence over the outcome. Responses provided are assumed to reflect both the participants’ information interpretation strategies as well as their information search strategies. Following a previous study investigating the association between causal illusion and the presence of paranormal beliefs, we expected that the association between causal illusion and pseudoscientific beliefs would disappear when controlling for the information search strategy (i.e., the proportion of trials in which the participants decided to present the potential cause). Volunteers with higher pseudoscientific beliefs also developed stronger causal illusions in active contingency detection tasks. This association appeared irrespective of the participants with more pseudoscientific beliefs showing (Experiment 2) or not (Experiment 1) differential search strategies. Our results suggest that both information interpretation and search strategies could be significantly associated to the development of pseudoscientific (and paranormal) beliefs.

## Introduction

The term “epistemically unwarranted beliefs” [1] refers to beliefs endorsed in the absence of substantial evidence supporting them (e.g., believing in ghosts or astral journeys, in the therapeutic benefits of Bach flowers, or that “chemtrails” contain biological agents sprayed to psychologically control the population). Although they lack adequate scientific support, these kinds of beliefs are relatively common in western society: 40% of the European population believes in lucky numbers [2], and 37% of the U.S. population considers astrology to be scientific [3]. Several studies have investigated the conditions favouring the presence of unwarranted beliefs, mostly focusing on those related to the paranormal [4–8]. Nevertheless, their possible cognitive basis is still unclear.

Causal illusion has been proposed as one possible cognitive phenomenon underlying unwarranted beliefs. The term causal illusion refers to the erroneous impression of a causal relationship between two unrelated events [9]. This cognitive bias can be induced experimentally in the context of a contingency detection task (e.g., [10]). One way of doing this is by asking the volunteers to judge the extent to which two events, a candidate cause and an outcome, are related (e.g., drug intake and healing from a health condition). In an *active* version of this experimental task (e.g., [11]), in each trial, participants are allowed to decide whether or not to introduce the potential cause (e.g., administer the drug to a patient). Immediately afterwards, they are informed whether the outcome appears (e.g., whether the patient has recovered) or not. After a pre-specified number of trials, volunteers are asked about the causal relationship between the events (causal rating). For instance, they are asked to indicate the level of effectiveness of the medicine, which they should typically rate on a scale from 0 (ineffective) to 100 (totally effective). This measure is taken as an indicator of the degree of perceived causal relationship and has been extensively used in previous studies (e.g., [10–14]). The relative densities of the different combinations of events will indicate the level of contingency between medicine administration and cure. In causal illusion tasks, these densities are manipulated so that the cure is not contingent on the administration of the medicine [i.e., the probability of recovery is equal whether the medicine is present or absent, P(Cure|Medicine) = P(Cure|¬Medicine)]. Therefore, the higher the causal ratings, the stronger the developed causal illusion is considered to be.

Importantly, since participants in *active* contingency detection tasks are given the opportunity to manipulate the presence or absence of the potential cause in order to explore its possible influence over the outcome, responses provided in this kind of task are assumed to reflect the participants’ information search strategies, i.e., how they look for new information in order to generate a causal impression; as well as their information interpretation strategies, i.e., how they integrate given information to generate a causal impression [15]. In this sense, as suggested by Griffiths, Shehabi, Murphy, and Le Pelley [15], superstitious individuals might be characterized by a general bias leading them to overweight conjunctive events, that is, cases in which cause and outcome (e.g., drug intake and healing) occur together, relative to disjunctive events, that is, when cause and outcome do not co-occur. This general bias could be expressed either in a tendency to actively search for conjunctive events (e.g., frequently administering the drug) or in a tendency to overestimate the relevance of conjunctive events (e.g., cases in which the drug is administered and healing occurs) when inferring the strength of the causal connection, or both. Nevertheless, so far, it remains unclear to what extent information search and information interpretation strategies have a role in the association between causal illusion and unwarranted beliefs.

To our knowledge, three studies have investigated the relationship between causal illusion and endorsement of these kinds of beliefs. First, Blanco, Barberia, and Matute [16] investigated the relationship between causal illusions and the development of paranormal beliefs. These authors presented participants with an *active* contingency learning task framed in a medical scenario in which they had to decide whether or not to administer a fictitious drug to patients suffering a fictional disease. In reality the drug was ineffective, as the probability of healing remained at 0.75 whether the drug was administered or not (their design also included a contingent task, but we will focus on the non-contingent one for our purposes here). After the volunteers provided their causal rating, they were asked to complete a questionnaire measuring several paranormal beliefs. Blanco *et al*. [16] observed a significant positive correlation between endorsement of paranormal beliefs and causal ratings provided in the contingency learning task. They also found that the amount of trials in which the participants administered the drug was positively associated both with causal ratings and with the score on the paranormal beliefs scale. Crucially, this search tendency fully mediated the correlation between causal ratings and paranormal beliefs. This observation led them to suggest that believers in the paranormal might be characterized by biased information-sampling strategies, which would make them more susceptible to develop erroneous causal impressions.

Blanco *et al*.’s [16] results contrast with those obtained by Griffiths *et al*. [15], who investigated the association between causal illusions and superstitious beliefs by means of an *active* contingency learning task framed in a non-medical scenario. These authors asked their volunteers to determine the extent to which the illumination of a lightbulb depended on pressing a switch. Switch-pressing and illumination were non-contingent, as the lightbulb illuminated about 60% of the time regardless of whether or not the switch was pressed. Once the participants provided their causal rating at the end of the task, they were asked to respond to a questionnaire measuring superstitious beliefs. Griffiths *et al*. [15] showed that the presence of superstitious beliefs correlated positively with the level of causal illusions developed in their contingency learning task. Importantly, although the task used by Griffiths *et al*. [15] was designed as an *active* contingency learning task, the interpretability of their results in relation to the impact of information search strategies over the development of unwarranted beliefs is limited because the authors instructed their participants to press the switch in about half of the trials. This was done in an attempt to control, to a certain extent, the times that participants exposed themselves to the potential cause (i.e., pressing the switch) with the aim of ensuring that the volunteers experienced enough cause-present and cause-absent trials. While this manipulation made sense for their study, it also implies that although the volunteers were, in principle, free to press the switch or not, their information search behaviour was constrained by the instructions. In this context, Griffiths *et al*.’s [15] results suggest that variability in the way one interprets given information, and not just the way we look for new information, could also be playing a role in the development of unwarranted beliefs.

Both Blanco *et al*.’s [16] and Griffiths *et al*.’s [15] studies were aimed at investigating the possible association between causal illusion and paranormal/superstitious beliefs. More recently, a third study has tried to extend those results to the directly related, but conceptually distinct, field of (also unwarranted) pseudoscientific beliefs. The term pseudoscience refers to disciplines which are presented as scientific knowledge but do not qualify as such [17], while paranormal and superstitious beliefs refer to phenomena that would contradict basic principles of science if they were true [18]. In addition, paranormal and pseudoscientific beliefs differ in their prevalence. For instance, while 22.7% of the Spanish population believes in paranormal phenomena, the percentages increase for pseudoscientific treatments such as homeopathy, 52.7%, and acupuncture, 59.9% [19]. Regarding pseudoscientific beliefs, Torres, Barberia, and Rodríguez-Ferreiro [20] used a *passive* contingency detection task framed in terms of a natural remedy. In this kind of task, volunteers are passively presented with different combinations of presence or absence of the cue and outcome events (e.g., remedy and relief). Torres *et al*. [20] observed a positive correlation between causal ratings given on the contingency learning task and scores on a scale designed *ad hoc* to measure the presence of pseudoscientific beliefs. Their results indicate an association between endorsement of pseudoscientific beliefs and causal illusion. Nevertheless, given that information sampling was not allowed by their design, their results are not informative with respect to the influence of information search strategies in that association.

In the present study, we aim to extend Torres *et al*.’s [20] results by further investigating the nature of the association between causal illusion and unwarranted beliefs. To this end, we presented participants with a measure of pseudoscientific beliefs and asked them to complete a contingency learning task in which they were free to decide whether to introduce the potential cause or not (i.e., an *active* task). A limitation of Torres *et al*.’s study was that they framed the task in the context of testing the efficacy of a natural remedy. Given the possible consideration of this remedy as a pseudotherapy, it could have been the case that volunteers with higher levels of pseudoscientific beliefs might have been more inclined to believe in the natural remedy irrespective of the contingency observed during the task. Taking this into account, we decided to use a neutral (i.e., non-pseudoscientific) scenario (the light bulb illumination scenario used by Griffiths *et al*. [15]) to frame our experiment. In line with Torres *et al*. [20], our hypothesis is that the strength of causal illusion will be associated with endorsement of pseudoscientific beliefs. Moreover, and in consonance with Blanco *et al*.’s [16] observations, we expect that this association will vanish when considering individual differences in the participants’ search strategy in situations in which they are free to decide how to look for causal information.

## Experiment 1

### Method

#### Participants

A total of 112 psychology students from the University of Barcelona participated in this experiment. Ninety-six were women and 16 were men, with ages ranging from 20 to 57, and a mean of 22.29 years old (*SD* = 4.25). The study protocols were approved by the ethics committee of the University of Barcelona (Institutional Review Board IRB00003099, Comissió de Bioètica de la Universitat de Barcelona). The study was performed in a regular class of the Psychology degree. The students could decide, at the end of each task, if they wanted to consent for their data to be used anonymously for research purposes or not. We obtained the participants’ written consent as follows: They were presented with the consent statement on the screen and they had to tick a box if they agreed. Only the data from students who gave consent are presented. Due to the COVID-19 global pandemic, we were forced to stop on-campus testing for this experiment. After adapting the task into an on-line version, we kept on testing participants. The results corresponding to the full (on-line and on-campus) sample, which are consistent with those obtained with the on-campus sample, are presented as S1 File.

#### Materials

*Contingency task*. Our contingency learning task, based on that designed by Griffiths *et al*. [15], was framed in a neutral scenario. The volunteers were required to judge the control that a switch (i.e., cause) had over the illumination of a light bulb (i.e., outcome). Specifically, initial instructions stated that their task was to find out whether a switch controlled the illumination of a light bulb. They were told that the electrical installation was old and very complicated, and that the switch and the bulb were separated from each other, so they had to test the switch and then go see if the bulb had turned on or not. They were also informed that there may have been other switches in other parts of the building that controlled the same bulb. Finally, they were further informed that the light bulb had a timer and turned off some time after it had been turned on, and that, once turned off, the switch could be tested again.

The participants had a total of 48 trials to explore the relation between these two events. In each trial, on a computer screen, they had the image of an unlit light bulb and a switch, and they were asked whether they wanted to press the switch. The participants had to click on a tick or a cross, depending on their decision. Then, the feedback appeared on the screen with either the light bulb on and the sentence "The light bulb has gone on!" or the bulb off and the sentence "The bulb is still off". The outcome (i.e., light bulb illumination) occurred following two randomized sequences, one for each decision. Specifically, it happened 6 out of every 8 trials, both among trials in which participants decided to press the switch and among those in which they chose not to. Therefore, the switch did not control the illumination of the light bulb, as illumination rates were non-contingent on the decision to press the switch, i.e., P(Outcome|Cause) = P(Outcome|¬Cause) = 0.75. After completing all 48 trials, the participants were required to provide a causal rating (i.e., “To what extent do you think the switch controls the bulb? Please use the sliding scale to respond. You can click inside the scale as many times as you wish until you mark the value you deem most appropriate. Any value between 0 and 100 is valid”). The value of zero was labelled as “No control”, and the value of 100 was labelled as “Total control”.

*Pseudoscience Endorsement Scale*. We gathered responses to the Pseudoscience Endorsement Scale (PES, [20]) from 97 participants (81 women and 16 men, mean age 22.30, *SD* = 4.48). The scale comprises 20 items referring to popular pseudoscientific myths and disciplines. Participants’ responses to each item were provided on a Likert-like scale ranging from 1 (i.e., “Totally disagree”) to 7 (i.e., “Totally agree”). Higher scores on this measure mean that the participants show greater endorsement of pseudoscientific beliefs.

*Superstitious Beliefs Questionnaire*. Following Griffiths *et al*. [15], we also included the Spanish version of their Superstitious Beliefs Questionnaire (SBQ, [20]), a translated version of the original English questionnaire by Griffiths *et al*. [15], as a complementary measure to the PES. We gathered responses to this questionnaire from 106 participants (93 women and 13 men, mean age 22.34, *SD* = 4.35). The volunteers had to rate 25 statements on a scale from 0 (i.e., “Strongly disagree”) to 4 (i.e., “Strongly agree”). Higher scores on this questionnaire indicate that the participant presents a higher level of superstitious beliefs.

#### Procedure

The participants first completed the computerized contingency learning task followed by the PES [20] and the SBQ [15], in that order. The two questionnaires were presented through Qualtrics (http://www.qualtrics.com).

### Results

The dataset employed in the analysis is available at https://osf.io/f4jcx/?view_only=afb95c269c00499b96b6cdf3423b95e4. We used JASP (version 0.16.0.0) to carry out all data analysis. The Bayesian *t*-tests were conducted using JASP’s default Cauchy prior width, *r* = 0.707. The Bayes factors (*BF*) were interpreted following Table 1 in Wagenmakers *et al*. [21], according to which values above 1, 3 and 10 indicate, respectively, anecdotal, moderate and strong evidence favouring the alternative (*BF*_*10*_) or the null (*BF*_*01*_) hypothesis.

Concerning the contingency task, since we let each participant decide how many times they pressed the switch or not, there was the possibility that some of them experienced a contingency slightly different from zero. Thus, we calculated the individual contingency (experienced ΔP) between switch pressing and bulb illumination experienced by each participant. In order to ensure that only data from participants who experienced a contingency close to 0 entered the analysis, we identified outliers (i.e., three *SD* above or below the mean) on the experienced ΔP, leading to the removal of one case. In addition, we also removed participants who always or never introduced the potential cause (11 participants) because such approach does not allow them to determine whether cause and outcome are related or not (i.e., if participants were only exposed to the probability of the outcome with or without the potential cause, the experienced contingency is not computable). The resulting sample consisted of 100 participants (87 women and 13 men, mean age = 22.30, *SD* = 4.44).

In relation to the questionnaires, both the PES and the SBQ showed high reliability, α = 0.91 and α = 0.92, respectively. The scores obtained on the PES, *mean* = 3.32 (in a 1 to 7 scale), *SD* = 0.96, were higher than those obtained on the SBQ, *mean* = 1.08 (in a 0 to 4 scale), *SD* = 0.68. We tested the correlations by means of Kendall’s tau, since the Shapiro-Wilk test showed that neither the causal ratings, *W*(99) = 0.94, *p* < .001, or the scores on the SBQ, *W*(94) = 0.97, *p* = .025, followed a normal distribution. Scores on both questionnaires were positively correlated, *r*_*τ*_ = 0.49, *p* < .001, *BF*_*10*_ = 1.150e+8.

**Fig 1:**
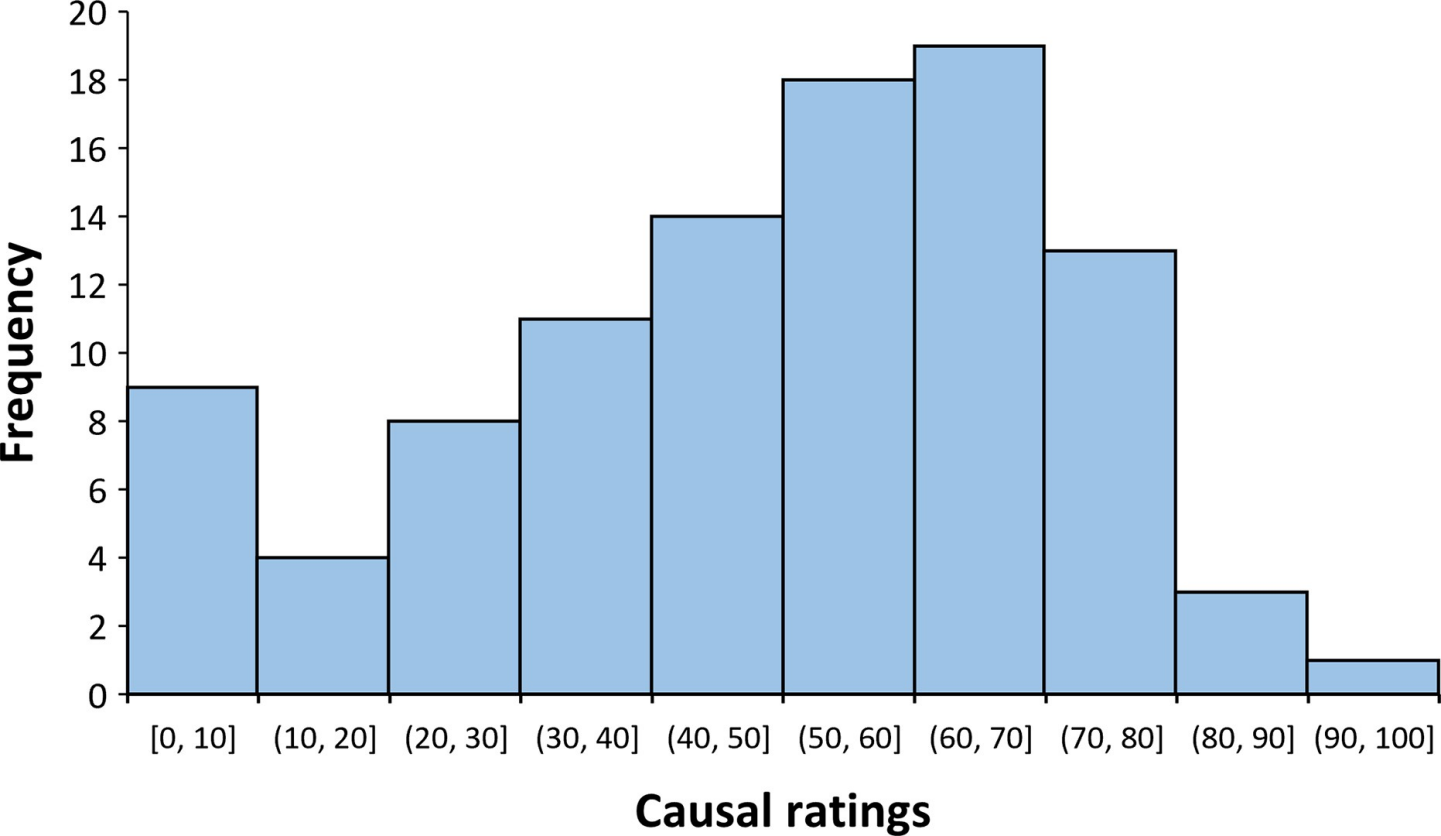
Distribution of causal ratings in Experiment 1.

**Fig 2:**
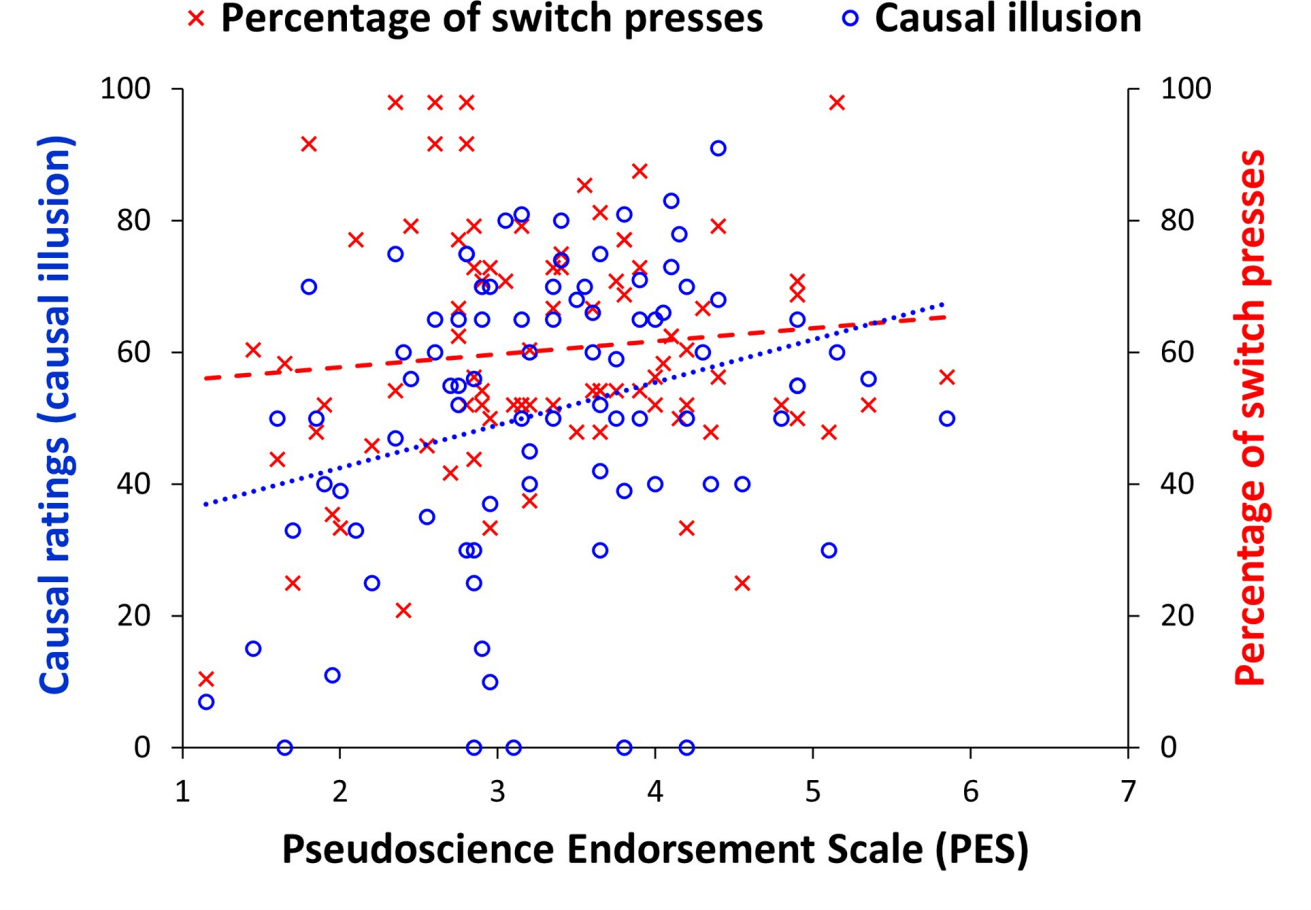
Scatterplot showing the associations between the main variables in Experiment 1.

Fig 1 shows the distribution of causal ratings in the contingency learning task (*mean* = 50.35, *SD* = 22.64). Fig 2 shows the association between mean scores on the PES and both the causal ratings (i.e., causal illusion) and the percentage of switch presses (*mean* = 0.60, *SD* = 0.18). We observed a positive correlation between percentage of switch presses and causal ratings, *r*_*τ*_ = 0.41, *p* < .001, *BF*_*10*_ = 7.095e+6, between causal ratings and scores on the PES, *r*_*τ*_ = 0.17, *p* = .021, *BF*_10_ = 2.17, and between causal ratings and scores on the SBQ, *r*_*τ*_ = 0.24, *p* < .001, *BF*_10_ = 48.25. In contrast, we observed no significant correlations between percentage of switch presses and scores on the PES, *r*_*τ*_ = 0.04, *p* = .625, and scores on the SBQ, *r*_*τ*_ = 0.14, *p* = .059. The Bayesian analogue analyses showed moderate, *BF*_*01*_ = 6.32, and anecdotal, *BF*_01_ = 1.16, evidence favouring the null hypothesis, for the correlations of switch presses with the PES and the SBQ, respectively. Next, we repeated some of the previous correlational analyses, while controlling for the individually experienced contingency (partial correlations) on the contingency learning task, in order to control for subtle deviations from zero in the experienced contingency that could explain the observed associations between causal ratings in the contingency learning task and the rest of the variables. The previous conclusions were corroborated as, even when controlling for the experienced ΔP, causal ratings remained significantly associated with scores in PES, *r*_*τ*_ = 0.17, *p* = .019, SBQ, *r*_*τ*_ = 0.24, *p* < .001, and the percentage of switch presses, *r*_*τ*_ = 0.40, *p* < .001.

Previous studies have shown that the percentage of cases in which the potential cause is present affects the intensity of causal illusions: the higher the percentage of cause-present trials the stronger the causal illusion developed [11]. Thus, we conducted a partial correlation between causal ratings and scores on the PES, controlling both for the experienced contingency and for the percentage of switch presses, which was statistically significant, *r*_*τ*_ = 0.17, *p* = .021. An analogous partial correlation between causal ratings and the scores on the SBQ, again controlling for both the experienced contingency and the percentage of switch presses, also reached significance, *r*_*τ*_ = 0.21, *p* = .004. These results suggest that the correlation between causal ratings and mean scores on both questionnaires is robust enough to remain even when the percentage of switch presses is controlled.

Analyses carried out without eliminating any participant led us to the same conclusions as those presented above, with only one exception: The correlation analysis between causal ratings and mean scores on PES approached, but did not reach, significance, *r*_*τ*_ = 0.123, *p* = .080, *BF*_*01*_ = 1.554.

### Discussion

The results of experiment 1 confirmed the association between endorsement of pseudoscientific (and superstitious) beliefs and the tendency to develop causal illusions observed in previous studies [20, 15]. This effect appeared even though the causal illusion task was framed in a neutral (non-pseudoscientific) scenario and the participants were free to decide whether or not to introduce the cause throughout the task. In relation to this, and in conflict with results by Blanco *et al*. [16], the volunteers’ information search strategy was not associated with the presence of unwarranted beliefs. Hence, the association between causal illusion and belief endorsement did not disappear when controlling for cause introduction rate.

When trying to reconcile the results of Experiment 1 and previous data by Blanco *et al*. [16] we came up with two possible explanations. One possibility is that information search strategies are differentially associated with different types of unwarranted beliefs. In their study, Blanco *et al*. [16] applied the Revised Paranormal Beliefs scale by Tobacyk (RPBS, [22]), which is one of the most common scales used to measure endorsement of paranormal beliefs. In contrast, we used the PES, aimed at measuring endorsement of pseudoscientific beliefs, and the SBQ, aimed at measuring superstitious beliefs (usually considered a subtype of paranormal beliefs). Note, however, that a close inspection of this last scale reveals that it includes both items related to paranormal beliefs (e.g., “I am interested in learning more about paranormal activity or psychic phenomena”), but also to pseudoscientific beliefs [e.g., “It is possible to gain information about a person’s personality by analysing their handwriting”, or “’Alternative’ therapies (such as homeopathic remedies, aromatherapy, reflexology, chiropractic manipulation, or therapy based on the body’s energy fields) can be an effective way of treating illnesses and ailments.”]. Thus, it could be the case that the data gathered in our experiment did not adequately reflect endorsement of paranormal beliefs, and, hence, the lack of influence of information search strategies is specific to the association between causal illusion and pseudoscientific beliefs.

A second possibility is related to the framing of the tasks used by Blanco *et al*. [16] and in our Experiment 1. Blanco *et al*. [16] framed their task as a medical scenario, in which the participants had to ascertain whether a given treatment was effective as a cure for a medical condition, as opposed to the more neutral (light bulb illumination) scenario used in our case. Previous studies have shown that causal illusions are facilitated by certain information search strategies [23, 24]. Specifically, participants develop stronger causal illusions when applying a confirmatory search strategy, consisting of a spontaneous tendency to test the relationship between the two events mainly observing cases in which the potential cause is present [11]. In this sense, it could be the case that the medical and neutral scenarios differentially led the volunteers to get involved in active confirmation of the tested hypothesis (i.e., the medicine heals the condition vs. the switch controls the light bulb).

With these two hypotheses in mind, we conducted a second experiment in which we attempted to replicate Blanco *et al*.’s [16] experiment including both the RPBS and the PES as measures of paranormal and pseudoscientific beliefs respectively. Following Blanco *et al*., the scenario we employed for the contingency learning task was medical. However, note that, while they presented a scenario in which a fictitious drug was to be tested as a remedy against a fictitious disease, in our case the drug was presented as a potential remedy for headaches. If search strategy is differentially associated with paranormal and pseudoscientific beliefs, then we could expect that the relation between causal illusion and paranormal beliefs might disappear when controlling for the tendency to introduce the candidate cause during the task. In contrast, the association between causal illusion and pseudoscientific beliefs would remain even when controlling for this factor. On the other hand, if the lack of effect of information search strategies in our previous experiment was due to the use of a more neutral scenario, then we could expect the search strategy to impact the association between causal illusion and endorsement of both paranormal and pseudoscientific beliefs.

Furthermore, the second experiment also incorporated two additional measures, i.e., an intelligence test and a question regarding the education level of the participants. We included them in order to evaluate if any of these potentially confounding factors could explain the previously found association between causal illusions and unwarranted beliefs.

## Experiment 2

### Method

#### Participants

A total of 190 participants were recruited through the on-line experimentation platform Prolific (https://www.prolific.co/) for this study. Half of the volunteers were women and the other half were men. Their mean age was 31.53 years old (*SD* = 11.02), ranging from 18 to 82. The study protocols were approved by the ethics committee of the University of Barcelona (Institutional Review Board IRB00003099, Comissió de Bioètica de la Universitat de Barcelona). The volunteers were presented with the consent statement on the screen at the beginning of the experiment and they agreed to participate in the study by entering their Prolific ID.

#### Materials

*Contingency learning task*. The volunteers were asked to determine the extent to which an experimental medicine (i.e., cause) was effective as a treatment for headache (i.e., outcome). Specifically, instructions indicated that they had to imagine that they were studying the extent to which an experimental medicine was effective as a treatment for headache, and that they would be shown several medical records of patients suffering a headache episode.

Over 40 trials, for each patient, their task was to decide whether or not to administer the medicine during the headache episode. In each trial, they had three seconds to decide whether they wanted to administer the medicine, in which case they had to click on the image of a pill, or not to administer it, in which case they just had to wait for the three seconds to pass without doing anything. Then, they received feedback about whether or not the patient overcame the headache within two hours and they were moved on to the next record. The medicine was not effective against the headache, as the rates remained non-contingent also for this experiment: P(Outcome|Cause) = P(Outcome|¬Cause) = 0.75, following the same sequences as in Experiment 1 (i.e., 6 out of every 8 trials the patient recovers from the headache, both when the medicine was administered and when it was not). After the 40 trials, the volunteers were asked to give a causal rating (i.e., “To what extent do you think the experimental medicine is effective as a cure for headache? Answer using the following scale, where the numbers are interpreted as follows: 0: Not effective at all; 100: Totally effective.”).

*Pseudoscientific Beliefs Scale (PES)*. The same scale measuring endorsement of pseudoscience used in Experiment 1 was also introduced in this experiment.

*Revised Paranormal Beliefs Scale (RPBS)* [22]. We used the RPBS to measure the level of endorsement of paranormal beliefs. Participants were presented with 26 statements that they had to rate on a Likert-like scale ranging from 1 (“Strongly disagree”) to 7 (“Strongly agree”). The level of endorsement of paranormal beliefs is the mean of the responses to each item, with higher scores indicating stronger paranormal beliefs.

Given the presumably greater heterogeneity of the sample recruited for Experiment 2 (general population through an online recruitment platform) compared with Experiment 1 (psychology students), we also included additional measures aimed to assess two variables which have been previously associated with variability in unwarranted belief endorsement: intelligence and level of education [25].

*Raven’s Advanced Progressive Matrices (APM-I)* [26]. Volunteers were presented with this twelve-item scale designed to measure general intelligence. Each item consists of a drawing matrix, presented in black ink on a white background, which is missing a part. The participants had to choose one of eight given options to complete the matrix, and the complexity of the matrices increased as the items passed through. The score on the questionnaire was calculated as the sum of correct responses, with higher values indicating higher general intelligence.

*Education level*. In order to get a measure of the education level of the participants, we introduced a question asking them to state how many years they had been in formal education, with indications ranging from “Primary or Elementary education: total of approximately 6 years” to “PhD: total of approximately 22 years”.

The three questionnaires were presented through Qualtrics (http://www.qualtrics.com).

#### Procedure

The participants first completed the contingency learning task. Then, they responded to the PES [20] and the RPBS [22] in random order. Finally, they completed the APM-I [26] and indicated their years of schooling.

### Results

The dataset is available at https://osf.io/f4jcx/?view_only=afb95c269c00499b96b6cdf3423b95e4. Data analysis was analogous to that of Experiment 1.

An outlier analysis regarding the experienced ΔP resulted in the exclusion of four cases. Participants who always administered or never administered the medicine were also removed. Finally, the sample consisted of 181 participants (89 women and 92 men, mean age = 31.48; *SD* = 10.84). The same conclusions can be drawn from the analysis carried out without the elimination of any participant.

In relation to the unwarranted beliefs questionnaires, both the PES (*α* = 0.93) and the RPBS (*α* = 0.95) showed excellent internal consistency. In general, the mean scores on the PES (*mean* = 3.85, *SD* = 1.12) were higher than those gathered on the RPBS (*mean* = 3.32, *SD* = 1.34), both in a 0 to 7 scale, *t*(180) = 8.07, p < .001, *d* = 0.60, *BF*_*10*_ = 6.585e+10. Moreover, scores obtained on both scales were positively correlated, *r*_*τ*_ = 0.56, *p* < .001, *BF*_*10*_ = 8.408e+25.

**Fig 3:**
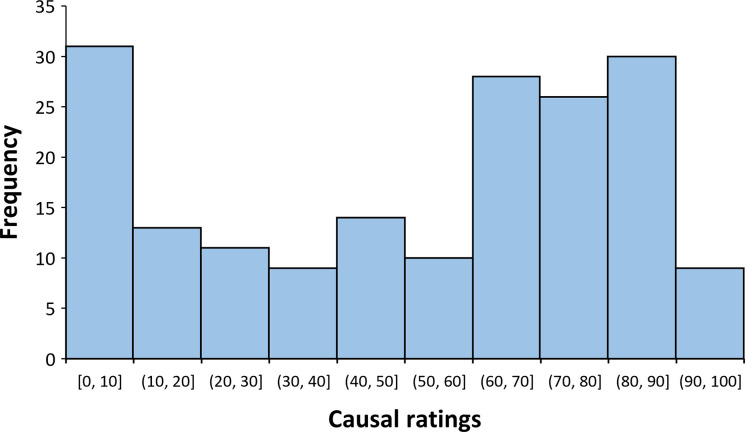
Distribution of causal ratings in Experiment 2.

**Fig 4:**
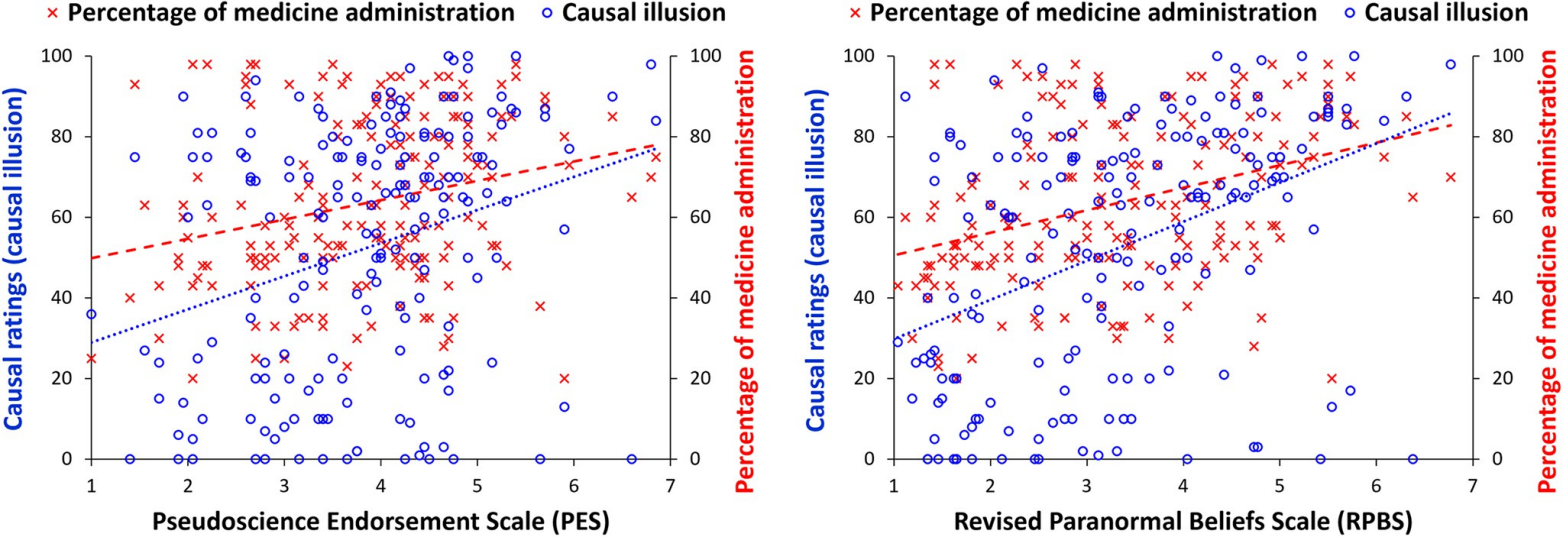
Scatterplots showing the association between the main variables in Experiment 2.

Fig 3 shows the distribution of causal ratings for Experiment 2 (*mean* = 52.39, *SD* = 31.07). Fig 4 shows the association between mean scores on the PES and mean scores on the RPBS, and both the causal ratings (i.e., causal illusion) and the percentage of medicine administration (*mean* = 0.64, *SD* = 0.21). All of them were positively correlated with each other: percentage of medicine administration and causal ratings, *r*_*τ*_ = 0.46, *p* < .001, *BF*_*10*_ = 1.201e+17; causal ratings and mean scores on the PES, *r*_*τ*_ = 0.21, *p* < .001, *BF*_*10*_ = 637.44; causal ratings and mean scores on the RPBS, *r*_*τ*_ = 0.31, *p* < .001, *BF*_*10*_ = 1.478e+7; percentage of medicine administration and mean scores on the PES, *r*_*τ*_ = 0.19, *p* < .001, *BF*_*10*_ = 96.34; and percentage of medicine administration and mean scores on the RPBS, *r*_*τ*_ = 0.26, *p* < .001, *BF*_*10*_ = 84190.90.

Next, and similar to Experiment 1, we replicated those previous correlational analyses that included causal ratings, while controlling for the individually experienced contingency (partial correlations). We also included other potential confounding factors that were measured in this experiment, i.e., the score of the participants in the Raven test (mean = 8.11, SD = 2.93) and the years of schooling (*mean* = 16.82, *SD* = 3.28). Causal ratings remained significantly associated with PES, *r*_*τ*_ = 0.15, *p* = .003, RBPS, *r*_*τ*_ = 0.25, *p* < .001, and percentage of medicine administration, *r*_*τ*_ = 0.42, *p* < .001, even when controlling for all these factors. Percentage of medicine administration also remained significantly associated both with PES, *r*_*τ*_ = 0.14, *p* = .007, and RPBS, *r*_*τ*_ = 0.21, *p* < .001.

Finally, and following the same assumption as in the previous experiment (i.e., the presence of pseudoscientific beliefs might be associated with both information interpretation strategies and information search strategies), we conducted a partial correlation between causal ratings and scores on the PES, controlling not only for the experienced contingency but, crucially, for the percentage of medicine administration, which returned a significant positive correlation, *r*_*τ*_ = 0.15, *p* = .003. The analogous analysis with the mean scores on the RPBS also showed a positive correlation, *r*_*τ*_ = 0.23, *p* < .001. Again, these results suggest that the correlation between causal ratings and mean scores on both questionnaires related to unwarranted beliefs (i.e., the PES and the RPBS) cannot simply be due to differences in the percentage of medicine administration.

## General discussion

Throughout this study, we examined the relationship between causal illusions and endorsement of unwarranted beliefs. In two experiments, our results revealed that volunteers with higher scores on different scales assessing pseudoscientific and paranormal beliefs tended to develop stronger causal illusions in a contingency learning task.

These results extend those reported by Blanco *et al*. [16], Griffiths *et al*. [15] and Torres *et al*. [20]. First, we replicated the association between endorsement of pseudoscientific beliefs and causal illusions generated in a contingency detection task, now framed in a neutral, non-pseudoscientific scenario. This observation suggests that the effect observed by Torres *et al*. [20] was not dependent on the use of a pseudoscientific cover and, hence, reinforces the hypothesis of the existence of a significant association between the tendency to develop causal illusions in simple contingency learning tasks and the endorsement of pseudoscientific beliefs. Moreover, we also observed an association between causal illusions and superstitious (SBQ) and paranormal (RPBS) beliefs, a result that is consistent with previous observations by Griffiths *et al*. [15] and Blanco *et al*. [16], respectively.

Torres *et al*. and Griffiths *et al*., respectively used a *passive* contingency learning task and an *active* task in which participants were instructed regarding how frequently they should introduce the potential cause. In contrast, volunteers in the present study were able to decide freely when to respond. This allowed us to investigate the role of spontaneous search strategies activated by the participants. As found in previous studies (e.g., [11, 24]), the participants’ tendency to introduce the potential cause in more trials was associated with the development of stronger causal illusions at the end of the task both in Experiments 1 and 2. Regarding the role of these search strategies in the association between causal illusions and unwarranted beliefs, our conclusions differ from those previously noted by Blanco *et al*. [16]. The association between causal illusions and unwarranted beliefs was meaningful even after controlling for the participants’ information search strategies. Specifically, in Experiment 1 we found no evidence of an association between the tendency to introduce the potential cause in the contingency learning task and the endorsement of neither pseudoscientific nor superstitious beliefs. In contrast, in Experiment 2 we did observe a positive correlation between the tendency to introduce the potential cause and the endorsement of pseudoscientific and paranormal beliefs, a result suggesting that individuals holding more unwarranted beliefs tend to search for causal information by more frequently introducing the potential cause. Noteworthily and differing from the results by Blanco *et al*. [16], the association between causal ratings in the contingency learning task and the scores in questionnaires measuring paranormal and pseudoscientific beliefs remained significant even when controlling for this behavioural component. This result suggests that believers might differ from nonbelievers, not only in their search strategies, but also in the way in which they interpret causal information.

Finally, although, as previously stated, paranormal and pseudoscientific beliefs differ both conceptually and in terms of prevalence [17–19], both types of beliefs positively correlated in our study, a result that is consistent with previous observations [1, 17, 20, 27]. Furthermore, our data suggest that they might both share a common cognitive tendency to develop causal illusions, since both types of unwarranted beliefs produced the same associations with causal illusions.

The divergent results between the two experiments regarding the association between search strategies and unwarranted beliefs might be due to differences in the procedures applied in the contingency learning tasks of each of them. A significant source of divergence stems from the use of different cover stories. Whereas Experiment 1 asked participants to determine to what extent a switch controlled the illumination of a lightbulb, Experiment 2 was framed in a medical scenario where participants had to determine if an experimental drug was effective against headaches. As noted by a reviewer of a previous version of this manuscript, the medical scenario employed in the contingency task of Experiment 2 better aligns with the items included in the Pseudoscience Endorsement Scale (PES), some of which refer to remedies against medical conditions (e.g., “Homeopathic remedies are effective as complements in the treatment of some diseases”). In this sense, it might be the case that the association between search strategy and unwarranted beliefs is restricted to situations involving health-related issues. However, this account does not explain parallel results found regarding the Revised Paranormal Beliefs Scale (RPBS), where the items are not focused on medical treatments (e.g., “A person’s thoughts can influence the movement of a physical object”).

Differences in the description of each task could also be responsible for the discrepancies between the results of the two studies. In Experiment 1 the cover story explicitly stated that the outcome (i.e., lightbulb illumination) might be produced not only by the candidate cause (i.e., the specific switch under study) but also by other alternative causes (i.e., “[…] there may be other switches in other parts of the building that control the same bulb”). In contrast, in Experiment 2 the instructions did not mention any other tentative causes of the outcome (i.e., recovery from headache) apart from the candidate cause (i.e., the experimental drug). The fact that other possible causes were mentioned in the instructions of Experiment 1 might have reduced the strength of the general bias towards overweighting conjunctive events (for instance, by activating a secondary hypothesis regarding the possible connection between alternative causes and lightbulb illumination the participants would have tried to test by not pressing the switch), therefore, making the task less sensitive to detecting individual differences among search strategies. Indeed, although differences between cause administration rates of Experiments 1 (*mean* = 0.60) and 2 (*mean* = 0.64) did not reach significance, *t*(279) = -1.666, *p* = .097, *d* = -0.21, *BF*_*01*_ = 1.97, *variance* among them was larger in Experiment 2 (*SD* = 0.21) than in Experiment 1 (*SD* = 0.18) as a Levene’s test indicated, *F*(1,279) = 5.65, *p* = .018. It might have been the case that slight differences in the task instructions have led participants to engage in active search of the cause-outcome connection to a different extent. Increased variance between participants might have favoured the identification of a significant correlation between search strategy and unwarranted beliefs in Experiment 2. In any case, this explanation is merely tentative, and further studies should be conducted to ascertain whether explicit mention of alternative hypotheses influences the participants’ testing strategy.

This study is not without limitations. Following Griffiths *et al*. [15] we could hypothesize that the general bias leading individuals to overweight conjunctive events when assessing causal relations could be a facilitator for the acquisition and perseverance of unwarranted beliefs. This would explain that the same individuals showing high scores on questionnaires measuring previously acquired unwarranted beliefs also develop stronger causal illusions in our laboratory tasks. Nevertheless, our research is correlational and, hence, it does not allow extracting conclusions regarding the directionality of the association between sensitivity to causal illusions and proneness to holding unwarranted beliefs. Moreover, it could also be the case that third variables not included in our study are responsible for the observed association. In this sense, even though in Experiment 2 we controlled for some potential confounding variables, we cannot rule out this possibility, as a myriad of non-contemplated alternative variables might explain such correlation.

Another limitation refers to the scale used to measure the development of causal illusion in our contingency learning tasks. Although many previous studies have regularly relied on this type of causal or effectiveness rating [10–14], this measure is not without problems. In this sense, absolute scores on this scale are difficult to interpret, as it is not clear whether the participants are actually expressing the strength of the causal relation or if these ratings are influenced by other aspects, such as their confidence in the judgement [28]. Considering this, further studies should try to replicate our results including more directly interpretable dependent variables such as choice-related measures (see [29]).

Finally, the use of the term “causal illusion” in our study might be subject to discussion. Tasks investigating causal illusions have typically relied on contingency [30] as the normative statistic to which to compare causal impressions, and the terms “causal illusion” or “illusion of causality” have become the norm to denote the phenomenon of medium to high causal ratings in zero contingency contexts (e.g., [15, 31, 32]). Nevertheless, some authors have suggested that ratings that deviate from the programmed contingency should not necessarily be interpreted as errors or illusions, and have offered a rational explanation for the special importance given to conjunctive trials. Mckenzie and Mikkelsen [33] argued that, given certain assumptions, such as the rarity of the candidate cause and outcome events, it would be adequate from a Bayesian inference approach to consider conjunctive trials particularly informative. As noted by these authors, the assumption that the occurrence of each event is rare, that is, that their absence is more common than their presence, would not be restricted to the probabilities experienced in the contingency learning task, but might be the consequence of prior beliefs that participants carry to the lab.

In any case, even though the tendency to overweight conjunctive events when establishing causal relationships might be, to some extent, adaptive, our study suggests that it might also involve certain drawbacks, as indicated by the association between higher causal ratings in zero-contingency tasks and endorsement of paranormal and pseudoscientific beliefs in our life.

## Supporting information

S1 File Results corresponding to the full (on-line and on-campus) sample in Experiment 1. (DOCX)
