# Supplementary material for: Causal illusion in the core of pseudoscientific beliefs: The role of information interpretation and search strategies
Source: PLoS One. 2022 Sep 9;17(9):e0272201. doi: 10.1371/journal.pone.0272201 (PMC9462769; doi:10.1371/journal.pone.0272201)
Supplement: S1 File — (DOCX) [file pone.0272201.s001.docx]

## Experiment 1 (on-campus participants plus on-line extension)

## Participants of the on-line extension

The volunteers for the on-line extension of Experiment 1 were 92 Psychology students from the University of Barcelona (77 women and 15 men). Their average age was 23.39 years old (*SD* = 6.71), ranging from 20 to 70.

## Materials and Procedure of the on-line extension

First, the participants completed the contingency learning task. This task was an online adaptation of that used in Experiment 1, with the following differences. First, the participants had a total of 40 trials instead of 48. Second, in each trial, they indicated whether they wanted to press the button or not, by clicking on the image of a button with a finger or the image of a button alone, respectively. Third, the randomized sequences of outcomes were pre-programmed on a single general matrix, where 6 out of every 8 trials the bulb lit up, regardless of the decision of the participant.

After finishing with the contingency learning task, the participants responded to the PES [19] but, different from the on-campus version, they did not complete the SBQ.

## Results with the full sample (on-campus testing and on-line extension)

The dataset is available at <https://osf.io/f4jcx/?view_only=afb95c269c00499b96b6cdf3423b95e4>. All the participants (on-line testing and on-campus extension) were analysed together. The data analysis was analogous to that performed with the on-campus participants alone, which is reported as Experiment 1 in the main article.

The outliers’ analysis excluded three cases (1 from the on-campus testing and 2 from the on-line extension). Participants who always administered or never administered the medicine were also removed (11 from the on-campus testing and 3 from the on-line extension). Finally, the full sample consisted of 187 participants (160 women and 27 men; mean age = 22.61, *SD* = 5.27; ΔP mean = -0.01, SD = 0.10).

In relation to the PES questionnaire, its reliability was high for the experimental sample, α = 0.90 (*mean* = 3.27, *SD* = 0.91). S1 Fig shows the distribution of causal ratings in the contingency learning task (*mean* = 47.66, *SD* = 24.22). S2 Fig shows the association between mean scores on the PES and both the causal ratings (i.e., causal illusion) and the percentage of button presses (*mean* = 0.58, *SD* = 0.18). Shapiro-Wilk tests showed that causal ratings did not follow a normal distribution, *W*(186) = 0.93, *p* < .001. Thus, all correlations were tested by means of Kendall’s tau non-parametric test. Kendall correlation analysis showed a positive correlation between percentage of button presses and causal ratings, *r_τ_* = 0.36, *p* < .001, *BF_10_* = 3.726e+10, and between causal ratings and scores on the PES, *r_τ_* = 0.15, *p* = .004, *BF_10_* = 8.23. Critically, there was no significant correlation between the percentage of button presses and scores on the PES, *r_τ_* = 0.05, *p* = .355. The Bayesian analogue analysis showed moderate evidence favouring the null hypothesis, *BF_01_* = 6.48.


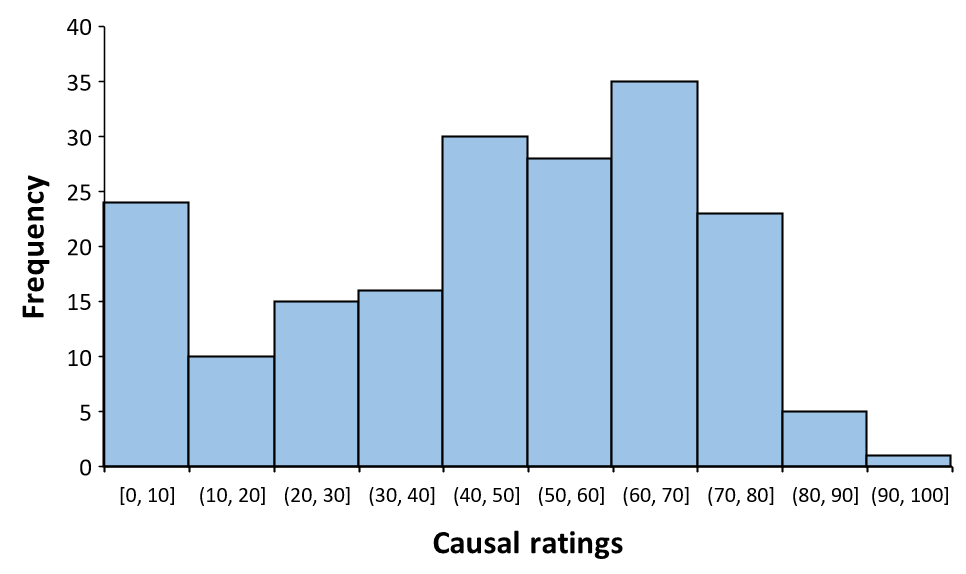


**S1 Fig. Distribution of causal ratings in Experiment 1 with the full sample.**


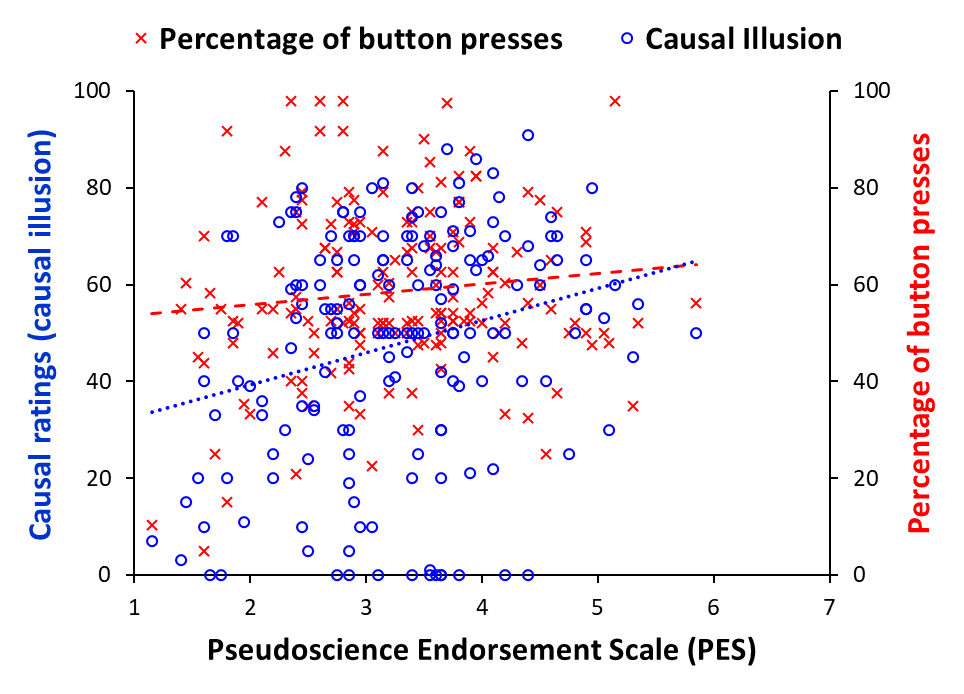


**S2 Fig. Scatterplot showing the association between the main variables in Experiment 1 with the full sample.**

Regarding the additional partial correlations, when controlling for the experienced ΔP, we found a positive correlation between causal ratings and scores in PES, *r_τ_* = 0.15, *p* = .003, and between causal ratings and the percentage of button presses, *r_τ_* = 0.36, *p* < .001. When controlling both for the experienced contingency and for the percentage of button presses, causal ratings and scores on the PES were also positively correlated, *r_τ_* = 0.14, *p* = .005.
